# Lectures on Syphilis

**Published:** 1875-11

**Authors:** 


Lectures on Syphilis. By Henry Lee, Professor of Surgery at Royal College of Surgeons of England. Published by Henry C. Lea, Philadelphia. Book; cloth; pp. 244.

This work is presented in the form of lectures, published for the first time, comprising ten. It is well arranged, typography neat, and contains ideas of practical interest. A brief extract from the preface will give some insight to the contents:

"The chief subjects treated of in the following lectures, which are not dwelt upon in the systematic works of other English authors of the present day, are, the inoculability of syphilitic blood in its various forms; the conditions under which the secretions of primary and secondary syphilitic manifestations may be inoculated naturally or artificially; the morbid processes produced by such inoculations; the modifications of those processes in patients previously syphilitic; primary and secondary syphilitic diseases of the mucous membranes, and their liability to communicate constitutional syphilis; the essential difference of the morbid processes in which the constitutional and local forms of syphilis respectively have their origin; and the pathology and treatment of discharges from the prostate gland, Cowper's glands, and the vesiculæ seminales."

